# Collapsin response mediator protein 4 isoforms (CRMP4a and CRMP4b) have opposite effects on cell proliferation, migration, and invasion in gastric cancer

**DOI:** 10.1186/s12885-016-2593-6

**Published:** 2016-07-30

**Authors:** Haijian Guo, Bing Xia

**Affiliations:** 1 Department of gastroenterology, Zhongnan Hospital of Wuhan University, Wuhan, Hubei 430071 People’s Republic of China; 2 Department of gastroenterology, the Second People’s Hospital of Shenzhen, Shenzhen, Guangdong, 518035 People’s Republic of China

**Keywords:** Collapsin response mediator protein 4, Gastric cancer, Opposite effects, Stable cells

## Abstract

**Background:**

Collapsin response mediator proteins (CRMPs) were originally identified in the nervous system and are involved in neuronal development. Similar to CRMP1, CRMP4 has a shorter transcript encoding a short isoform known as CRMP4a, and a longer transcript encoding a long isoform known as CRMP4b. Previous studies have shown that CRMP4a and CRMP4b exhibit opposing functions in neurite outgrowth. In the present study, we aimed to determine whether CRMP4a and CRMP4b have divergent effects in gastric cancer.

**Methods:**

We first analyzed the mRNA and protein expression levels of CRMP4a and CRMP4b in surgical resected specimens, gastric cancer cell lines and normal gastric epithelial cell line GES-1 by quantitative real-time PCR. Open reading frame and CRMP4b shRNA were generated by lentivirus package and stable cells stably expressing CRMP4a open reading frame and CRMP4b shRNA were constructed. Then the roles of CRMP4a and CRMP4b in cell proliferation, cell cycle progression, apoptosis, migration, invasion, and adhesion were determined by cell proliferation assays, flow cytometry analysis, transwell migration and invasion assays, cell Adhesion Assay, and tumorigenicity assays in nude mice, respectively.

**Results:**

CRMP4a expression was lower and CRMP4b expression was higher in tumor tissue samples as compared to paired non-tumor tissue samples. Additionally, CRMP4a expression was lower and CRMP4b expression was higher in gastric cancer cell lines than in the normal gastric epithelial cell line GES-1. CRMP4a overexpression and CRMP4b silencing suppressed cell proliferation in vitro and in vivo. Additionally, CRMP4a overexpression and CRMP4b silencing induced a significant G1-phase arrest and a decrease of the percentage of cells in S-phase. Furthermore, CRMP4a overexpression and CRMP4b silencing inhibited cell migration, invasion, and adhesion. However, neither CRMP4a overexpression nor CRMP4b silencing affected apoptosis.

**Conclusion:**

These results indicate that CRMP4a and CRMP4b have opposite effects on cell proliferation, migration, and invasion in gastric cancer.

## Background

Stomach cancer, also known as gastric cancer, is the third most common cancer [1]. Stomach cancer leads to 297,496 deaths annually, and the mortality rate is 22.08/100,000 [1]. Presently, treatment for stomach cancer may include surgery, chemotherapy, and/or radiation therapy [2–4]. However, none of these methods leads to a satisfactory reduction of the morbidity and mortality rates, because diagnoses are usually made after the disease has reached an advanced stage [5]. Thus, new treatment approaches, such as biological therapies, are needed to treat advanced stomach cancer. It is therefore necessary to identify critical targets in advanced stomach cancer in order to develop effective targeted treatments.

Collapsin response mediator proteins (CRMPs), also known as the dihydropyrimidinase-like protein (DPYSL) family, are cytosolic phosphoproteins that are highly expressed in the developing and adult nervous systems [6, 7]. The CRMP family consists of five homologous cytosolic proteins: CRMP1, CRMP2, CRMP3, CRMP4, and CRMP5 [8–11]. Although CRMPs were originally identified in the nervous system and are involved in neuronal development, previous studies have demonstrated that CRMPs are expressed in cancerous tissues and may affect cancer progression and metastasis [6, 12–14]. CRMP1 has a novel transcript variant that encodes a long isoform (LCRMP1) [15]. The functional difference between CRMP1 and LCRMP1 has previously been investigated in non-small-cell lung cancer (NSCLC) [16, 17] Low CRMP1 mRNA expression in lung cancer tissue was significantly associated with advanced disease, lymph node metastasis, early post-operative relapse, and shorter survival [17]. Thus, CRMP1 may function as a novel invasion suppressor gene in lung cancer. Conversely, expression of LCRMP1 mRNA was significantly higher in NSCLC tumor tissue than in adjacent normal tissues, and high LCRMP1 mRNA expression was associated with poor overall and disease-free survival in patients with NSCLC [18]. Collectively, these results show that LCRMP-1 and CRMP-1 have opposing functions in regulating cancer cell invasion and metastasis.

Similar to CRMP1, CRMP4 has one transcript variant encoding a short isoform known as CRMP4a, and a second transcript variant that encodes a long isoform known as CRMP4b. Previous studies have shown that CRMP4a and CRMP4b exhibit opposing functions in neurite outgrowth [6, 19]. Therefore, we hypothesized that CRMP4a and CRMP4b might exhibit opposing functions in regulating gastric cancer cell behavior. Our in vitro and in vivo results confirmed this hypothesis.

## Methods

### Patients and tissue samples

Thirty gastric cancer patients who underwent curative resection at Zhongnan Hospital of Wuhan University (Wuhan, China) were enrolled in the study. All pathological features were confirmed by experienced pathologists, and none of the patients received pre-operative anti-cancer treatment. Written informed consent for the use of resected tissues and participation in this study was obtained from all patients before surgery. The study was approved by the Institute Research Ethics committee of Zhongnan Hospital of Wuhan University.

### Cell lines and culture

The human gastric carcinoma cell lines BGC823, GC9811, HGC-27, MGC803, and NCI-N87as well as the human normal gastric epithelial cell line GES-1 were obtained from the Type Culture Collection of the Chinese Academy of Sciences (Shanghai, China). Cells were cultured in Dulbecco’s modified Eagle’s medium; HyClone, Logan, UT, USA) or RPMI-1640 (HyClone, Logan, UT, USA) containing 10 % fetal bovine serum (Gibco, Logan, UT, USA) and 100 U penicillin and streptomycin at 37 °C in a humidified atmosphere containing 5 % CO_2_.

### Lentivirus package and stable cells construction

The complete open reading frame (ORF) of CRMP4a (NM_001387.2) was amplified by polymerase chain reaction (PCR) using the primer pair 5′-CCGCTCGAG ATGTCCTACCAAGGCAAGAAGAAC-3′ and 5′-ATAAGAATGCGGCCGCTTACTTGTCATC-3′ containing XhoI and BamHI restriction sites within the 5′ and 3′ termini, respectively. CRMP4b shRNA was generated using the following single sequences: 5′-GATCCGAGGTTGGCTCTGACTGTATCAAGAGTACAGTCAGAGCCAACCTCTTTTTTTG-3′ and 5′-AATTCAAAAAAAGAGGTTGGCTCTGACTGTACTCTTGATACAGTCAGAGCCAACCTCG-3′. The amplicon was then inserted into the pLVX-IRES-ZsGreen1 plasmid. pLVX lentiviral particles containing CRMP4a ORF, and CRMP4b shRNA were generated by transiently transfecting 293 T cells. Lentivirus production, concentration, and titration were each performed according to standard procedures. For infection, 2 × 10^5^ MGC803 and BGC823 cells were divided into four groups and subcultured in 6-well culture plates for 24 h prior to transduction. The four groups of cells are as follows: MGC803 cells infected with negative control lentiviral suspension (MGC803-NC); MGC803 cells infected with lentiviral suspension expressing CRMP4a (MGC803-CRMP4a); BGC823 cells infected with negative control lentiviral suspension (BGC823-shNC); and BGC823 cells infected with lentiviral suspension expressing CRMP4b shRNA (BGC823-shCRMP4b). For transduction, the cell culture medium was removed and cells were washed twice with phosphate-buffered saline (PBS). Next, 0.5 mL of lentiviral suspension [1 × 10^8^ IU/mL, multiplicity of infection (MOI) =100] containing 8 μg/mL polybrene was added to the cells. Cells were then incubated at 37 °C overnight. The vector suspension was then aspirated from the cells, and transduced cells were added to 2 mL/flask fresh growth medium. Cells were then incubated at 37 °C in a humidified atmosphere containing 5 % CO_2_. Growth medium was replaced after 24 h. After allowing cells to incubate for 72 h, MGC803 and BGC823 cells were passaged twice per week with growth medium containing puromycin at a pre-determined dosage to select for cells expressing the transduced vector. Positively screened cell lines were sub-cloned three times using limiting dilution, then cultured in growth medium containing puromycin for one month to generate stable cell lines.

### RNA extraction and quantitative real-time PCR (qRT-PCR)

Total RNA was extracted from each MGC803 and BGC823 cell lineusing TRIzol reagent (Invitrogen, Carlsbad, CA, USA) according to the manufacturer’s protocol. RNA was reverse transcribed into cDNA using PrimeScript RT reagent kit with cDNA Eraser (Takara Bio, Dalian, China) in a 20 μL reaction according to the manufacturer’s protocol. Equal amounts of cDNA were used as templates for qRT-PCR to detect the level of CRMP4 and LCRMP4 expression relative to that of 18 s rRNA (endogenous control). mRNA expression was quantitated using an ABI PRISM 7500 Sequence Detection System (Foster City, CA, USA) and SYBR Green qPCR SuperMix (Invitrogen, Carlsbad, CA, USA). For CRMP4a, the forward and reverse primers were CRMP4a-F 5′-CATTCACTCCACCTGATCTC-3′ and CRMP4a-R 5′-CCCTCCTTCTTCTGCTCC-3′, respectively; for CRMP4b, the forward and reverse primers were CRMP4b-F 5′-GAAGACGATCTGCCCGTGTA-3′, CRMP4b-R 5′-AAATCCAGCGTCTTGCTCTC-3′, respectively; for 18 s rRNA, the forward and reverse primers were 18 s rRNA-F 5′-CCTGGATACCGCAGCTAGGA-3′ and 18 s rRNA-R 5′-GCGGCGCAATACGAATGCCCC-3′, respectively. qRT-PCR reactions were performed in duplicate and repeated three times. Fold induction of gene expression was calculated using the 2^−ΔΔCT^ method.

### Western blot analysis

MGC803 and BGC823 cells were washed twice with ice-cold PBS and resuspended in ice-cold RIPA buffer containing 1 mmol/L phenylmethanesulfonyl fluoride and a cocktail of protease inhibitors (1:100 dilution; Beyotime, Nantong, China). Samples were centrifuged at 4 °C for 15 min at 14,000 rpm. Supernatants were collected, and protein content was quantitated using a BCA Protein Assay kit (Thermo Scientific Pierce, Rockford, IL, USA). Equal amounts of protein were separated using 8–12 % SDS polyacrylamide gels, then transferred to PVDF membranes (Millipore, Billerica, MA, USA). Membranes were blocked for 1 h at 37 °C in blocking buffer (5 % milk in TBS containing 0.05 % Tween-20;TBST), then incubated with primary antibody (anti-CRMP4, 1:1,000; anti-Cyclin D1, 1:1500; anti-Cyclin E1, 1:2000; anti-Bcl2, 1:2000; anti-Caspase 3, 1:1500; anti-Caspase, 1:2000; anti-GAPDH, 1:2,000; Abcam, Cambridge, MA, USA) at 37 °C for 1 h. Membranes were then washed three times with TBST, incubated with horseradish peroxidase (HRP)-conjugated secondary antibody at 37 °C for 40 min, and washed three times with TBST before protein visualization using Immobilon Western Chemilum HRP Substrate (Millipore, Billerica, MA, USA). GAPDH served as an internal loading control. Densitometry analysis was performed on western blot images using Image Pro-Plus 6.0 software (Media Cybernetics, Silver Spring, MD, USA). To quantitate individual protein bands, a uniformly-sized square was drawn around each band to measure its density; the density value was then adjusted by the background density of a region near each band. The results of densitometry analysis were expressed as a relative ratio of the target protein to the reference protein. The relative ratio of target protein in the control group was set as 1.

### Cell proliferation assays

Cell proliferation was measured using the CellTiter 96 AQueous One Solution Cell Proliferation Assay kit (Promega, Madison, WI, USA) according to the manufacturer’s protocol. MGC803 and BGC823 cells (1 × 10^4^) cells were seeded onto a 96-well plate. After culturing for 0, 1, 2, and 3 days, 10 μL of CellTiter 96 AQueous One Solution reagent was added to each well. Cells were then incubated for 4 h at 37 °C. Absorbance was measured at 490 nm using a microplate reader (Multiskan MK3, Thermo Scientific, Vantaa, Finland). The proliferation rate was calculated using the following formula: proliferation rate = survival rate = (OD_test_/OD_negative control_) × 100 %.

### Flow cytometry analysis

Annexin V-FITC apoptosis detection and cell cycle detection kits were used to analyze the apoptosis rate and cell cycle distribution according to the manufacturer’s protocols (Keygen, Nanjing, China). Cells were dissociated using trypsin, then centrifuged at 2,000 rpm for 5 min. Next, cells were washed twice with PBS and centrifuged at 2,000 rpm for 5 min. For apoptosis analysis, the cell pellet (~1–5 × 10^5^ cells) was resuspended in 500 μL Binding Buffer. Then, 5 μL Annexin V-FITC and 5 μL propidiumiodide (PI) were added to the cell suspension, which was gently mixed and incubated at room temperature, protected from light, for 15 min. Within 1 h, the cells were analyzed via flow cytometry (BD Biosciences, San Jose, CA, USA). For cell cycle analysis, cells were fixed in 500 μL 70 % ice-cold ethanol at 4 °C overnight. Cells were then washed twice with 500 μL PBS. Up to 100 μL RNaseA was added and cells were incubated at 37 °C for 30 min. Next, 100 μL PI was added and cells were incubated at 4 °C in the dark for 30 min. The cell cycle distribution was then analyzed via flow cytometry (BD Biosciences, San Jose, CA, USA). Each experiment was repeated three times.

### Transwell migration and invasion assays

Cell migration and invasion were assessed using a transwell assay. For migration assays, MGC803 and BGC823 cells were harvested; then 1 × 10^5^ cells suspended in 100 μL of serum-free medium were placed in a transwell insert (pore size, 8 μm; BD Biosciences, San Jose, CA, USA). The lower chamber was filled with 600 μL medium containing 10 % FBS. For invasion assays, cells were suspended as in the migration assay, then placed into a transwell insert pre-coated with Matrigel (BD Biosciences, Bedford, MA, USA). After incubating cells for 24 h at 37 °C and gently removing the cells in the upper chamber with a cotton swab, the cells on the underside of the membrane were fixed with 4 % paraformaldehyde for 15 min, stained with 0.1 % crystal violet in 20 % ethanol, and counted in five randomly selected fields using phase contrast microscopy. Cells were imaged at 200× magnification using a Olympus microscope (Hamburg, Germany). Five independent fields per well were imaged. Each assay was performed in triplicate.

### Cell adhesion assay

Ninety-six-well dishes were pre-coated with 30 mg/L fibronectin solution (50 μL/well), then air-dried at room temperature overnight. Wells were rinsed with PBS and incubated with 3 % heat-denatured BSA to block any uncoated areas. Each group of MGC803 and BGC823 cells (1 × 10^5^/well) was seeded in coated wells and incubated for 2 h at 37 °C.Non-adherent cells were removed by washing the wells twice with PBS. DMEM containing10% FBS was added to each well, and cells were then incubated at 37 °C for 4 h. Next,10 μL of CellTiter 96 AQueous One Solution ((Promega, Madison, WI, USA)) was added to each well and cells were incubated for an additional 4 h at 37 °C. Absorbance was measured at 490 nm using a microplate reader (Multiskan MK3, Thermo Scientific, Vantaa, Finland).

### Tumorigenicity assays in nude mice

Six-week old male athymic nude mice were subcutaneously injected in the right armpit region with 4 × 10^6^ cells in 0.2 mL of PBS. Four groups of mice injected with MGC803-NC, MGC803-CRMP4a, BGC823-NC, and BGC823-shCRMP4b stable cells (*n =* 6/group) were tested. Tumor size was measured using calipers. Tumor volume was calculated using the formula (L × W^2^)/2, where L is the length and W is the width of the tumor. All experimental procedures involving animals were performed in accordance with the Guide for the Care and Use of Laboratory Animals (NIH publication no. 80–23, revised 1996) and approved by Institutional Animal Care and Use Committee of the Wuhan University.

### Statistical analysis

Statistical analyses were performed using SPSS19.0 software (IBM, Chicago, IL, USA). Results are depictedas mean ± standard deviation (SD). A Student’s *t*-test was used to compare means between groups. A *p*-value of less than 0.05 was considered statistically significant.

## Results

### CRMP4a and CRMP4bmRNA and protein expression in surgical specimens

**Fig. 1:**
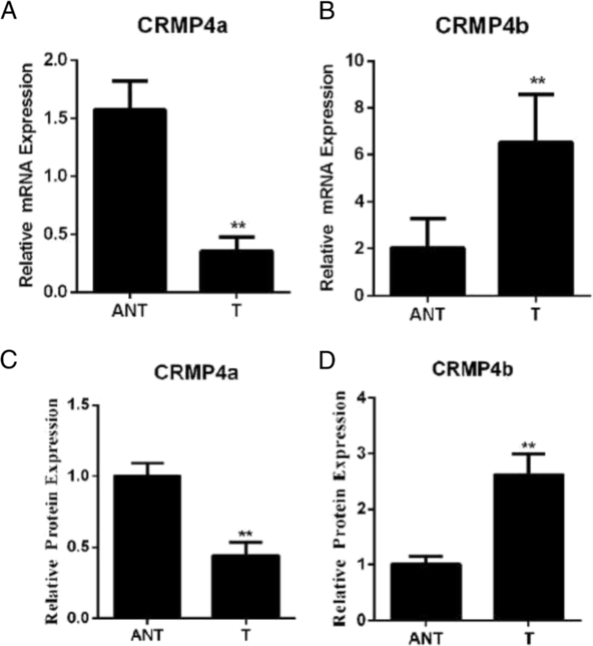
Expression levels of CRMP4a and CRMP4b in tumor and adjacent non-tumor (ANT) tissue samples. **a** CRMP4a mRNA expression level. **b** CRMP4b mRNA expression level. **c** Relative protein expression of CRMP4a. **d** Relative protein expression of CRMP4b. Expressed as mean ± SD. **p < .01

To examine CRMP4a and CRMP4b mRNA and protein expression levels, qRT-PCR and western blot analyses were performed on 30 pairs of surgical specimens (tumor and adjacent non-tumor tissue samples). A significant reduction in CRMP4a mRNA and protein expression was identified in tumor tissue samples as compared to paired non-tumor tissue samples (Fig. 1a). A significant increase in CRMP4b mRNA and protein expression was identified in tumor tissue samples as compared to paired non-tumor tissue samples (Fig. 1b). In addition, the correlations between the level of CRMP4a and CRMP4b mRNA between various clinicopathological parameters are analyzed and the results are summarized in Table 1. CRMP4a and CRMP4b mRNA expression was not found to be associated with age, or gender, while the decrease of CRMP4a expression and increase of CRMP4b were significantly associated with tumor diameter, depth of tumor invasion, distal metastasis, lymphatic metastasis and TNM stage (Table 1, *P* < 0.05 for each). These results indicate that decrease of CRMP4a expression and increase of CRMP4b may be associated with gastric cancer aggressiveness, especially tumor growth and invasion ability. Furthermore, a significant increase in CRMP4b mRNA and protein expression was identified in tumor tissue samples compared to paired non-tumor tissue samples (Fig. 1c, d).

**Table 1:** Correlation between CRMP4a and CRMP4b mRNA expression with the clinicopathological factors

Characteristic	CRMP4a			CRMP4b		
	n	Expression	P value	n	Expression	P value
Age			0.387			0.42
< 55	14	0.35 ± 0.05			5.26 ± 1.25	
≥ 55	16	0.30 ± 0.08			6.27 ± 2.03	
Gender			0.091			0.124
male	13	0.32 ± 0.05			6.29 ± 5.03	
female	17	0.41 ± 0.07			5.14 ± 3.43	
Tumor diameter			0.042			0.047
< 5 cm	18	0.29 ± 0.08			4.69 ± 2.69	
≥ 5 cm	12	0.15 ± 0.09			8.41 ± 2.94	
Depth of tumor invasion			0.012			0.025
T 1 + T 2	15	0.59 ± 0.12			3.51 ± 1.67	
T 3 + T 4	15	0.12 ± 0.07			9.21 ± 3.37	
Distal metastasis			0.011			0.015
M 0	20	0.61 ± 0.19			2.16 ± 1.58	
M 1	10	0.11 ± 0.02			8.97 ± 3.29	
Lymphatic metastasis			0.025			0.014
Negative	9	0.58 ± 0.13			3.09 ± 2.75	
Positive	21	0.17 ± 0.09			8.97 ± 2.29	
TNM stage			0.028			0.034
I-II	16	0.78 ± 0.25			4.25 ± 1.24	
III-IV	14	0.21 ± 0.12			9.58 ± 2.57	

### CRMP4a and CRMP4b mRNA and protein expression in gastric cancer cell lines and stable cell line construction

**Fig. 2:**
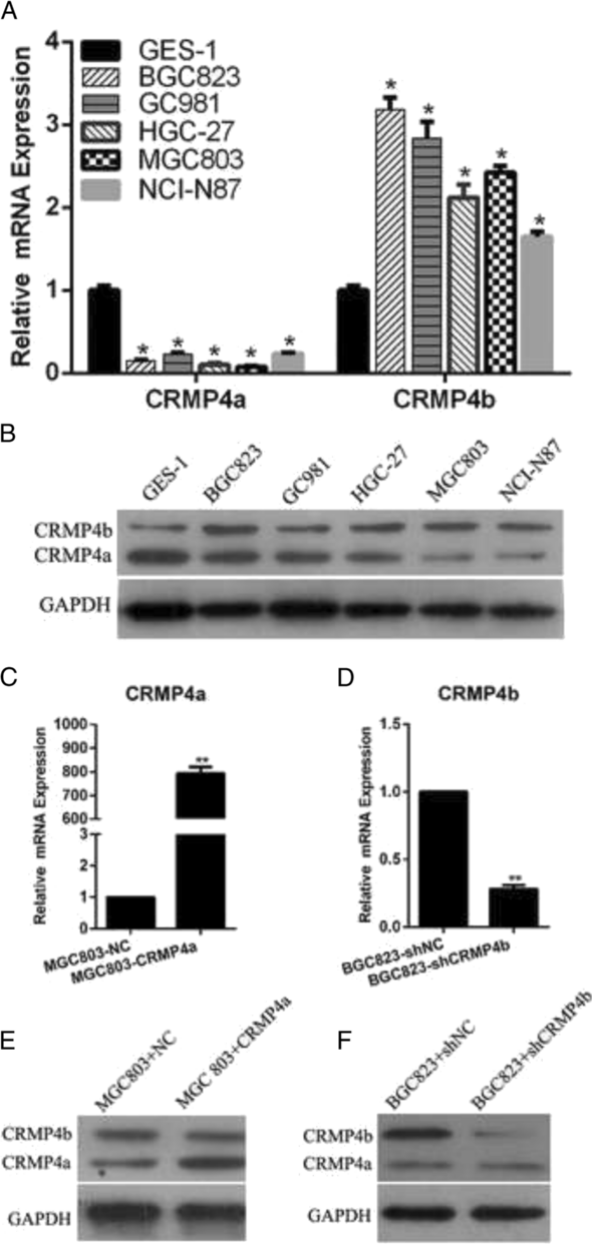
Expression levels of CRMP4a and CRMP4b in gastric cancer cell lines and stable cell lines. **a** The mRNA expression level of CRMP4a in gastric cancer cell lines. **b** The mRNA expression level of CRMP4b in gastric cancer cell lines. **c** The mRNA expression level of CRMP4a in stable cells. **d** The mRNA expression level of CRMP4b in stable cells. **e** The protein expression level of CRMP4a in stable cells. **f** The protein expression level of CRMP4b in stable cells. **p < .01

To further verify the mRNA and protein expression levels of CRMP4a and CRMP4b in gastric cancer, total RNA and protein was collected from human gastric carcinoma cells lines BGC823, GC9811, HGC-27, MGC-803, and NCI-N87, as well as from normal human gastric epithelial cell line GES-1. CRMP4a and CRMP4b mRNA and protein expression levels were then examined by qRT-PCR and western blot, respectively. The data show that CRMP4a mRNA and protein expression is reduced in gastric cancer cells as compared to normal gastric epithelial cells; CRMP4a mRNA and protein expression was lowest in MGC803 cells (Fig. 2a, b). CRMP4b mRNA and protein expression is increased in gastric cancer cells as compared to normal gastric epithelial cells; CRMP4b mRNA and protein expression was highest in BGC823 cells (Fig. 2a, b). Based on these data, we chose MGC803 and BGC823 cells for subsequent analyses.

To understand the function of CRMP4a and CRMP4b in gastric cancer, lentiviral particles expressing CRMP4a ORF, CRMP4b shRNA were packaged into lentiviral vectors and transduced into MGC803 or BGC823 cells at a MOI of 100. After puromycin selection, four transduced cell lines (MGC803-NC, MGC803-CRMP4a, BGC823-shNC and BGC823-shCRMP4b) were harvested for qRT-PCR and western blot analyses. Fig. 2b and c show that CRMP4a was successfully overexpressed at both the mRNA and protein levels in MGC803-CRMP4a cells. Similarly, CRMP4b was successfully silenced both at the mRNA and protein levels in BGC823-shCRMP4b cells (Fig. 2d and f). These four stable cell lines were thus determined to be sufficient for use in subsequent assays.

### CRMP4a overexpression or CRMP4b silencing suppressed cell proliferation in human gastric carcinoma cell lines

**Fig. 3:**
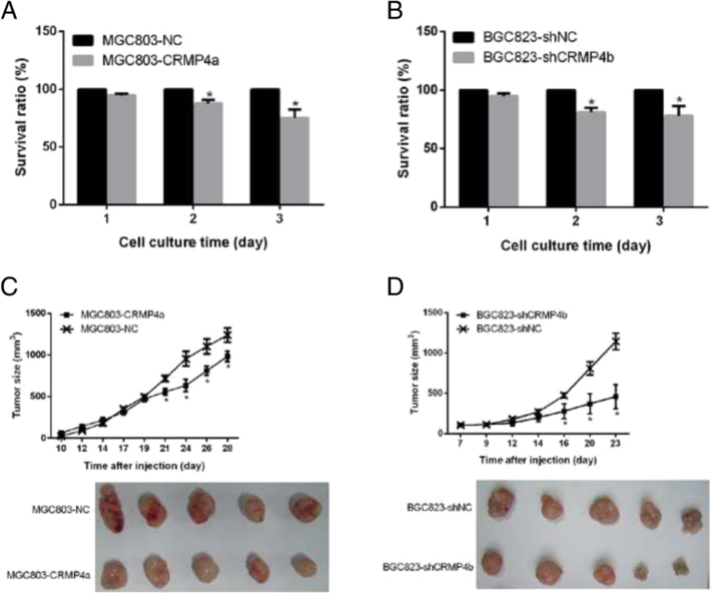
CRMP4a overexpression or CRMP4b silencing suppressed cell proliferation in human gastric carcinoma cell lines. **a** The effect of CRMP4a overexpression on MGC803 cell proliferation. **b** The effect of CRMP4b silencing on BGC823 cell proliferation. **c** Growth curves of tumors in nude mice (*above*); representative images of tumors isolated from nude mice (*below*). Nude mice were injected with MGC803-NC or MGC803-CRMP4a stable cells. **d** Growth curves of tumors in nude mice (*above*); representative images of tumors isolated from nude mice (below). Nude mice were injected with BGC823-shNC or BGC823-shCRMP4b stable cells. *p < .05

To determine whether CRMP4a overexpression or CRMP4b silencing affects cell proliferation, a cell proliferation assay was performed. CRMP4a overexpression significantly suppressed MGC803 cell proliferation on days 1 and 2 (Fig. 3a). Similarly, CRMP4b silencing significantly suppressed BGC823 cell proliferation on days 1 and 2 (Fig. 3b). To verify our in vitro findings, we examined the effects of CRMP4a overexpression and CRMP4b silencing on tumor growth in nude mice. Each of the four stable cell lines was injected subcutaneously into the flanks of nude mice. NC cells served as a control. We found that nude mice injected with cells overexpressing CRMP4a generated smaller tumors than those injected with NC cells (Fig. 3c). Similarly, nude mice injected with cells in which CRMP4b was silenced generated smaller tumors than those injected with shNC cells (Fig. 3d).

**Fig. 4:**
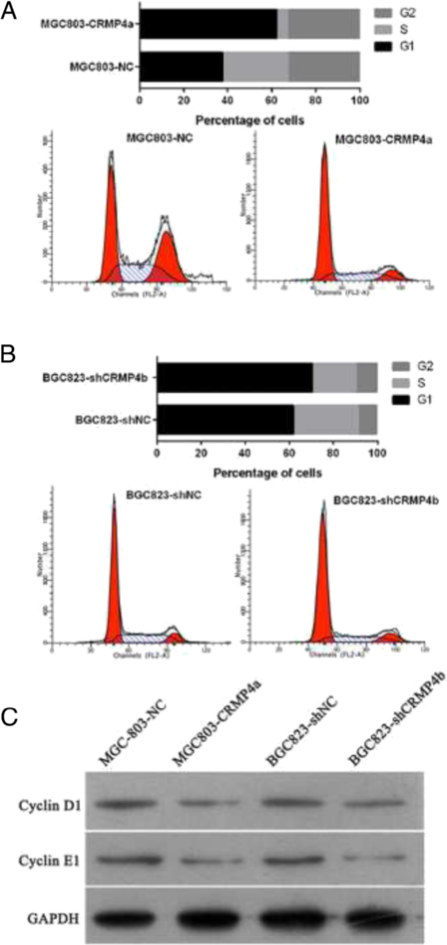
CRMP4a overexpression or CRMP4b silencing suppressed cell cycle in human gastric carcinoma cell lines. **a** The effect of CRMP4a overexpression on MGC803 cell cycle distribution. **b** The effect of CRMP4b silencing on BGC823 cell cycle distribution. **c** The effect of CRMP4a overexpression or CRMP4b silencing on the protein expression of Cyclin D1 and Cyclin E1

To determine the mechanism by which CRMP4a overexpression or CRMP4b silencing suppresses cell proliferation, we used flow cytometry to determine the distribution of cell cycle stages in each of the four stable cell lines. Over-expression of CRMP4a induced a significant G1-phase arrest in MGC803 cells (Fig. 4a, and the percentage of cells in S-phase decreased significantly. Similarly, CRMP4b silencing induced a significant G1-phase arrest in BGC823 cells (Fig. 4b), and the percentage of cells in S-phase decreased significantly. In addition, we also investigated the expression levels of Cyclin D1 and Cyclin E1 via western blot. CRMP4a overexpression or CRMP4b silencing decreased the protein expression of Cyclin D1 and Cyclin E1 (Fig. 4c).

### CRMP4a overexpression or CRMP4b silencing did not affect apoptosis in human gastric carcinoma cell lines

**Fig. 5:**
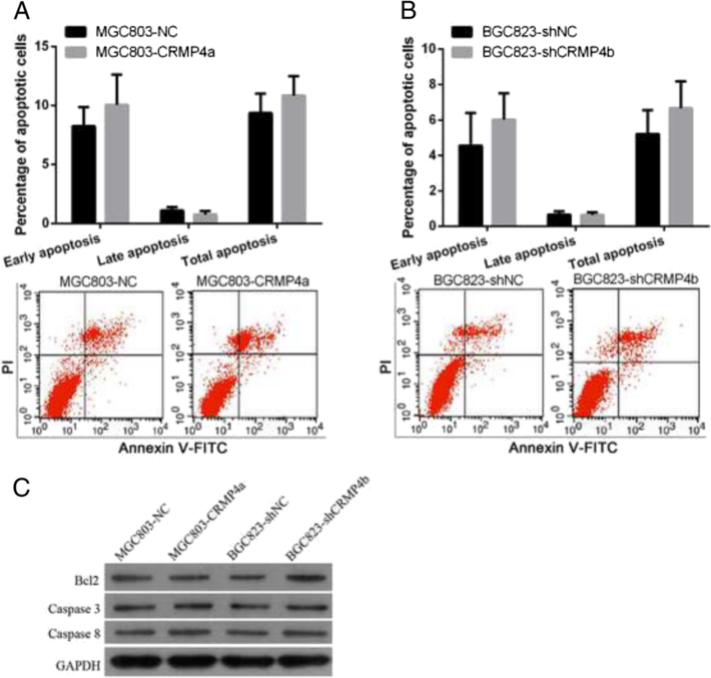
CRMP4a overexpression or CRMP4b silencing did not affect apoptosis in human gastric carcinoma cell lines. **a** The effect of CRMP4a overexpression on MGC803 cell apoptosis. **b** The effect of CRMP4b silencing on BGC823 cell apoptosis. **c** The effect of CRMP4a overexpression and CRMP4b silencing on the protein expression levels of Bcl2, Caspase 3, and Caspase 8

Next, we investigated the effects of CRMP4a overexpression and CRMP4b silencing on apoptosis using flow cytometry analysis. CRMP4a overexpression did not significantly affect the percentage of apoptotic MGC803 cells (Fig. 5a. We also investigated the expression levels of apoptosis-related proteins via western blot. CRMP4a overexpression did not affect the protein expression of Bcl2, Caspase 3, or Caspase 8 (Fig. 5b). Similarly, CRMP4b silencing did not affect the percentage of apoptotic cells or the protein expression of Bcl2, Caspase 3, or Caspase 8 in BGC823 cells (Figs. 5b, c).

### CRMP4a overexpression or CRMP4b silencing inhibited cell migration, invasion, and adhesion in human gastric cancer cell lines

**Fig. 6:**
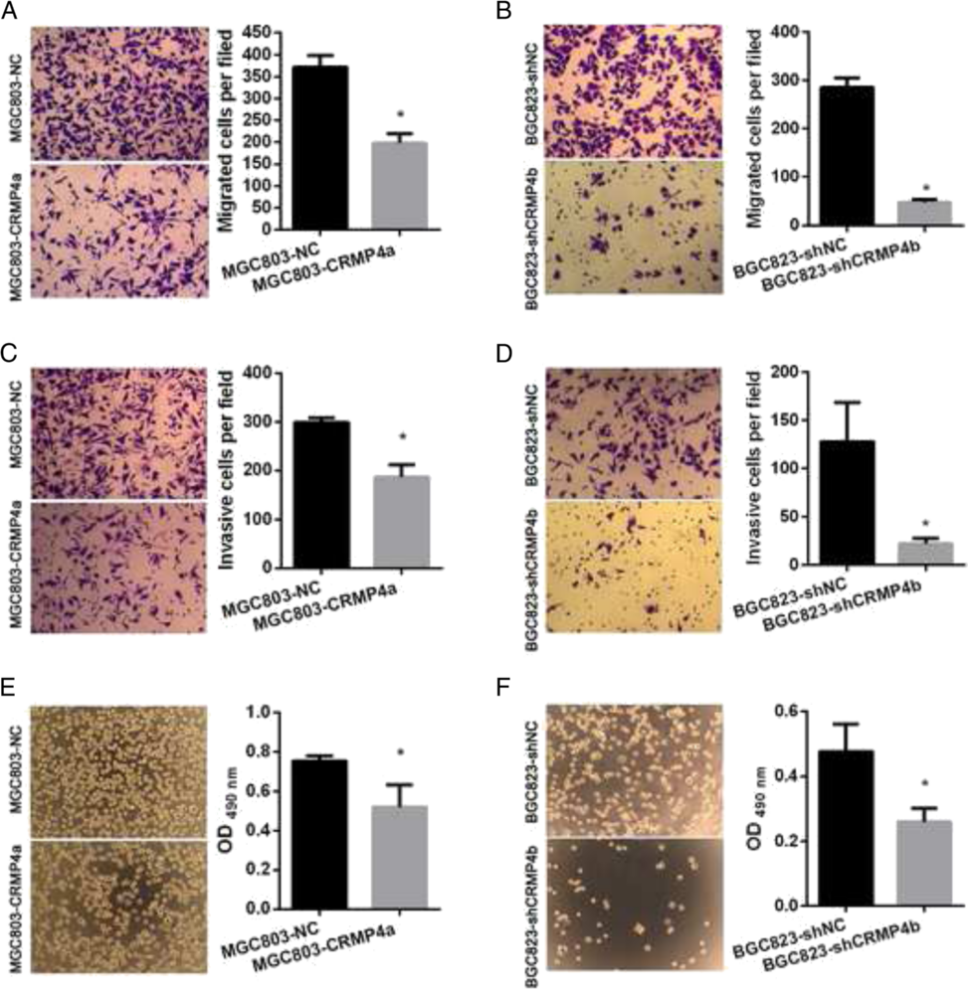
CRMP4a overexpression and CRMP4b silencing inhibited cell migration, invasion and adhesion. **a** The effect of CRMP4a overexpression on MGC803 cell migration. **b** The effect of CRMP4b silencing on BGC823 cell migration. **c** The effect of CRMP4a overexpression on MGC803 cell invasion. **d** The effect of CRMP4b silencing on BGC823 cell invasion. **e** The effect of CRMP4a overexpression on MGC803 cell adhesion. **f** The effect of CRMP4b silencing on BGC823 cell adhesion

To determine the role of CRMP4a and CRMP4b in regulating human gastric cancer cell migration, invasion, trans-well cell migration and invasion assays were performed. Adhesion assays were similarly performed to determine the role of CRMP4a and CRMP4b in regulating human gastric cancer cell adhesion. For trans-well migration assays, the number of cells that passed through the membrane into the lower chamber was significantly lower in MGC803 cells overexpressing CRMP4a than in NC MGC803 cells (Fig. 6a). Similarly, the number of cells that passed through the membrane into the lower chamber was significantly lower in BGC823 cells in which CRMP4b was silenced than in shNC BGC823 cells (Fig. 6b. For trans-well invasion assays, the number of cells that passed through a Matrigel-coated membrane into the lower chamber was significantly lower in MGC803 cells overexpressing CRMP4a than in NC MGC803 cells (Fig. 6c). Similarly, the number of cells that passed through a Matrigel-coated membrane into the lower chamber was significantly lower in BGC823 cells in which CRMP4b was silenced than in shNC BGC823 cells (Fig. 6d). The adhesion ability of CRMP4a-overexpressing MGC803 cells was significantly weaker than that of NC MGC803 cells (Fig. 6e). Similarly, the adhesion ability of BGC823 cells in whichCRMP4b was silenced was significantly weaker than that of shNC BGC823 cells (Fig. 6f).

## Discussion

CRMP4 isoforms, also named as TUC-4a and TUC-4b, were firstly identified in nervous system and palyed a role in regulating neurite outgrowth and were associated with vesicles in the growth cone [19]. Furthermore, CRMP4a (TUC-4a) and CRMP4b (TUC-4b) exhibit opposing functions in neurite outgrowth [6, 19]. In addition, altered CRMP4 expression has been observed in several malignant tumors, including prostate cancer, pancreatic cancer, and neuroblastoma [14, 20–22]. In prostate cancer, CRMP4 expression was inversely associated with lymph node metastasis [14]. Furthermore, CRMP4 overexpression not only suppressed the invasion ability of prostate cancer cells *in vitro*, but also strongly inhibited tumor metastasis in an animal model [14]. These data implicate CRMP4 as a metastasis suppressor in prostate cancer [14]. In pancreatic cancer, CRMP4 mRNA and protein expression was significantly increased. Moreover, CRMP4 knockdown using siRNA reduced invasion, but did not affect proliferation [22]. These data implicate CRMP4 as a metastasis promoter in pancreatic cancer. The opposing effects ofCRMP4 expression on prostate and pancreatic cancer metastasis may reflect a difference in the CRMP4 splice variant that is predominantly expressed [6]. In the present study, we comprehensively investigated the function of the short isoform, CRMP4a, and the long isoform, CRMP4b, on gastric carcinoma cell behavior for the first time.

In the present study, we investigated the function of CRMP4a and CRMP4b in gastric carcinoma cell proliferation, cell cycle progression, migration, invasion, and adhesion. We found that CRMP4a overexpression and CRMP4b silencing suppressed gastric carcinoma cell proliferation, migration, cell cycle progression, invasion, and adhesion, but did not affect apoptosis. These results differ from those of prior studies on prostate and pancreatic cancer: CRMP4 had no effect on cell proliferation in either of these cancers [20, 22]. Consistent with the results of previous studies on prostate and pancreatic cancer, we found that CRMP4a and CRMP4b play an important role in regulating cell migration and invasion. Based on these results, we hypothesize that CRMP4 has different effects on different cancer cell types.

Tumorigenesis can proceed as a result of altered cell cycle progression, leading to uncontrolled cellular proliferation [23]. In the present study, we found that CRMP4a overexpression and CRMP4b silencing induced a significant G1-phase arrest and a decrease in the percentage of cells in S-phase. CRMP4a overexpression and CRMP4b silencing also suppressed cell proliferation. We thus conclude that abnormal cell cycle progression induced by CRMP4a overexpression or CRMP4b silencing is a potential cause of tumor growth suppression.

Our data indicate that CRMP4a acts as a tumor suppressor in gastric carcinoma. This conclusion is supported by several of our findings: (1) CRMP4a expression was reduced in gastric carcinoma tissue samples and gastric carcinoma cell lines; (2) CRMP4a overexpression suppressed cell proliferation in vitro and in vivo; (3) CRMP4a overexpression inhibited cell migration, invasion, and adhesion in human gastric carcinoma cell lines; and (4) CRMP4a overexpression inhibited cell migration in vivo. In addition, our data indicate that CRMP4b acts as an oncoprotein in gastric carcinoma. This conclusion is also supported by several of our findings: (1) CRMP4b expression was increased in gastric carcinoma tissue samples and gastric carcinoma cell lines; (2) CRMP4b silencing suppressed cell proliferation in vitro and in vivo; (3) CRMP4b silencing inhibited cell migration, invasion, and adhesion in human gastric carcinoma cell lines; and (4) CRMP4b silencing inhibited cell migration in vivo. Collectively, these results show that CRMP4a and CRMP4b have opposing functions in regulating gastric carcinoma cell proliferation, invasion, and metastasis. Our results are supported by a previous study that revealed CRMP4a and CRMP4b to exhibit opposing functions in neurite outgrowth [6, 19].

## Conclusion

Understanding the opposing functions of CRMP4 isoforms in regulating gastric cancer cell proliferation, migration, and invasion.provides new insights into the role of CRMP4 in these processes. All our results indicated that CRMP4 might be a new biomarker for the diagnosis and prognosis of gastric cancer patients and might be a new target for advanced gastric cancer.

## Abbreviations

ANT, adjacent non-tumor; CRMPs, Collapsin response mediator proteins; MOI, multiplicity of infection; NSCLC, non-small-cell lung cancer; PBS, phosphate-buffered saline; qRT-PCR, qantitative real-time PCR
